# Temporal Bone Osteosarcoma in Pregnancy: A Case Report and Literature Review

**DOI:** 10.1055/a-2979-5191

**Published:** 2026-10-20

**Authors:** Andrea M. Asaf-Calderon, Jesica Z. Sandoval-Ocampo, Salvador I. Victor-Ramos

**Affiliations:** 1 Department of Obstetrics and Gynecology 37768 National Institute of PerinatologyMexico CityCDMXMexico; 2 Department of Neonatology 37768 National Institute of PerinatologyMexico CityMexico; 3 Department of Oncology 37768 National Institute of PerinatologyMexico CityMexico

**Keywords:** osteosarcoma, pregnancy, chemotherapy, fetal growth restriction

## Abstract

**Introduction** Osteosarcoma is the most common primary malignant bone tumor; however, involvement of the temporal bone is exceptionally rare, particularly during pregnancy. The coexistence of malignancy and pregnancy presents unique diagnostic and therapeutic challenges requiring multidisciplinary management.

**Case Presentation** A 23-year-old woman presented with persistent right otalgia and an external auditory canal lesion initially diagnosed as cholesteatoma. Following surgical resection, final histopathology revealed temporal bone osteosarcoma with early locoregional recurrence and intracranial extension. Pregnancy was incidentally diagnosed during oncologic evaluation at 24 weeks of gestation. Given the aggressive nature of the tumor, pregnancy termination followed by palliative chemotherapy was initially recommended; however, the patient elected to continue the pregnancy after multidisciplinary counseling. She received chemoradiotherapy with ifosfamide/mesna and 40 Gy radiotherapy. Subsequently, severe anhydramnios and early fetal growth restriction developed, likely secondary to fetal nephrotoxicity associated with ifosfamide exposure. A cesarean section was performed after antenatal corticosteroid administration, resulting in the delivery of a live preterm neonate admitted to the neonatal intensive care unit, and discharged after 58 days of hospitalization.

**Conclusion** Temporal bone osteosarcoma during pregnancy is exceedingly rare and requires complex multidisciplinary management balancing maternal oncologic treatment and fetal risks.

## Introduction

Cancer in pregnancy is one of the most distressing conditions in obstetrics and gynecology, occurring in around 1:1000 pregnancies with an increase in incidence due to the recent tendencies in delaying childbearing. Diagnosis of oncological conditions during pregnancy may be challenging given that tumor symptoms can be masked by normal physiologic changes of pregnancy. 1 Cancer in pregnancy is defined as a cancer that occurs during pregnancy or in the first year postpartum, and constitutes the second leading cause of mortality in women of reproductive age. 2 The most frequent cancers associated with pregnancy are breast, lymphoma, cervix, leukemia, ovary, gastrointestinal, thyroid, and melanoma. 3 Adverse pregnancy outcomes can be associated with either the diagnosis or with the surgical, pharmacological, or radiological treatment offered. Placental metastases are rare, with melanoma being the most common cause (30% of reported placental metastasis), and metastases to the fetus are even rarer, which arise when cancer cells breach the intervillous space. 4

Primary bone tumors are rare, accounting for approximately 15% of all sarcomas and less than 0.2% of all malignancies. 5 Among them, osteosarcoma is the most common subtype, typically occurring in children and adolescents. It is characterized by a high risk of recurrence and metastasis, and is associated with a poor overall prognosis. 6

Osteosarcoma presents more frequently in men, and has a bimodal fashion, with the first group being 13–16 years old. 7 The highest incidence is seen in regions such as Africa, South Asia, and Central and South America. 8 Long bones are the most frequently affected sites, with approximately 75% of cases involving the distal femur. 7 There is a second peak in incidence among adults in their seventh and eighth decades of life, particularly in those over 65 years old. In this population, the tumor is often considered secondary to another condition (such as Paget’s disease, prior radiotherapy, or other disorders). 9 Although it remains more frequent in men than in women, its incidence is higher in Caucasians compared with other racial groups. In this age group, the most common sites are the axial skeleton and areas previously exposed to radiation or with preexisting bone abnormalities. Metastatic involvement at the time of diagnosis is also more frequent, and the 5-year survival rate is lower than in younger patients. 6 10

Pathogenesis of this disease is multifactorial, mainly due to altered mesenchymal cell differentiation, disruption of tumor suppressor gene function, activation of oncogenic pathways, epigenetic modifications, and increased cytokine production. 11 Genetic mutations seen in osteosarcoma include RB1 and TP53. 12 Main risk factors for osteosarcoma are radiation therapy during the first years of life, with an average interval between exposure and development of 12–16 years, although it may be shorter the earlier the exposure occurs, or in case of exposure to certain alkylating agents as chemotherapy in pediatric cancers, Paget’s disease, especially in older patients, which is associated with worse prognosis, and chronic bone lesions, such as chronic osteomyelitis, bone infarcts, and benign bone tumors. 13 14

Pediatric patients have better outcomes than adults, with adults experiencing higher relapse rates and lower OS and EFS. For example, in large registry and cooperative group studies, adults (≥18 years) have 5-year OS rates ranging from 41% to 46%, compared with 60% to 71% in pediatric patients. 13 15

## Case Presentation

A 23-year-old woman with no significant past medical or family history presented to the ENT department in February 2025 with a 7-month history of persistent pain in the right earlobe and external auditory canal, initially noted in July 2024. Over the preceding weeks, she developed swelling in the external auditory canal, prompting further evaluation. On physical examination, a lesion was identified in the canal and subsequently biopsied, with initial pathology reporting a cholesteatoma, after which the patient underwent tumor resection via a middle cranial fossa approach with right canaloplasty.

However, final histopathology revealed an osteosarcoma of the middle cranial fossa with unknown surgical margins. The patient was referred to an oncology center, where imaging less than 1 month postoperatively demonstrated locoregional recurrence with clinical and imagenologic unresectable criteria. During routine laboratory workup, a positive serum pregnancy test was discovered. Obstetric ultrasound was performed, showing a fetal biometry of 24.3 weeks with an estimated weight of 602 ± 101 g (9th percentile, Hadlock), normal amniotic fluid, and a fundal placenta.

Given the aggressive nature of the tumor, initial recommendations included pregnancy termination followed by systemic palliative chemotherapy with doxorubicin and cisplatin. After multidisciplinary counseling, the patient decided to continue the pregnancy; therefore, palliative chemoradiation for local tumor control was given, consisting of ifosfamide/mesna combined with 40 Gy of radiotherapy ( Fig. 1 ). Afterwards, the patient reported decreased fetal movements; during ultrasonographic evaluation, anhydramnios was reported, the reason for which she was referred to a tertiary hospital for further obstetric and maternal fetal evaluation, where an ultrasound evaluation confirmed anhydramnios. Crystallization and PAMG-1 determination tests were performed, ruling out premature membrane rupture as cause of anhydramnios. The patient was admitted for close maternal and fetal monitoring, with serial complete blood count (CBC), C-reactive protein (CRP), and erythrocyte sedimentation rate (ESR; Fig. 2 ) and twice daily non-stress test (NST). Serial laboratory tests showed no signs of systemic infection. Assessment by the genetics department concluded that the fetal diagnoses of early-onset fetal growth restriction and anhydramnios were most likely due to the history of initiation of chemoradiotherapy with ifosfamide/mesna at 24.3 weeks of gestation, consisting of 15 cycles with a total dose of 40 Gy in 15 fractions, stating that pharmacovigilance studies have shown that exposure to ifosfamide between 16 and 30 weeks of gestation is associated with oligohydramnios, fetal growth restriction, and neonatal acute renal failure secondary to fetal nephrotoxicity. 16 As the pregnancy was considered viable and had both fetal growth restriction and anhydramnios, a multidisciplinary meeting was held among the obstetrics, oncology, and neonatology departments. It was decided to proceed with an elective abdominal delivery. Antenatal corticosteroids were administered, as well as fetal neuroprotection with magnesium sulfate for a total of 9 h prior to resolution. A cesarean section was performed, resulting in the birth of a live newborn weighing 970 g and measuring 35.5 cm in length, with a Ballard score consistent with 29 weeks of gestation, Apgar scores of 7 and 9, and a Silverman–Anderson score not assessable. The neonate was subsequently transferred to the neonatal intensive care unit (NICU) for further management and monitoring. Maternal postoperative recovery was adequate, and the patient was discharged and referred to an oncology center with a brain MRI ( Figs. 3 and 4 ) to continue management. The MRI showed right temporal mass extending through the external auditory canal into the intracranial compartment, with infiltration of the temporal lobe and associated vasogenic edema, as well as areas of cystic degeneration and/or central necrosis, consistent with the referring diagnosis.

**Fig. 1:**
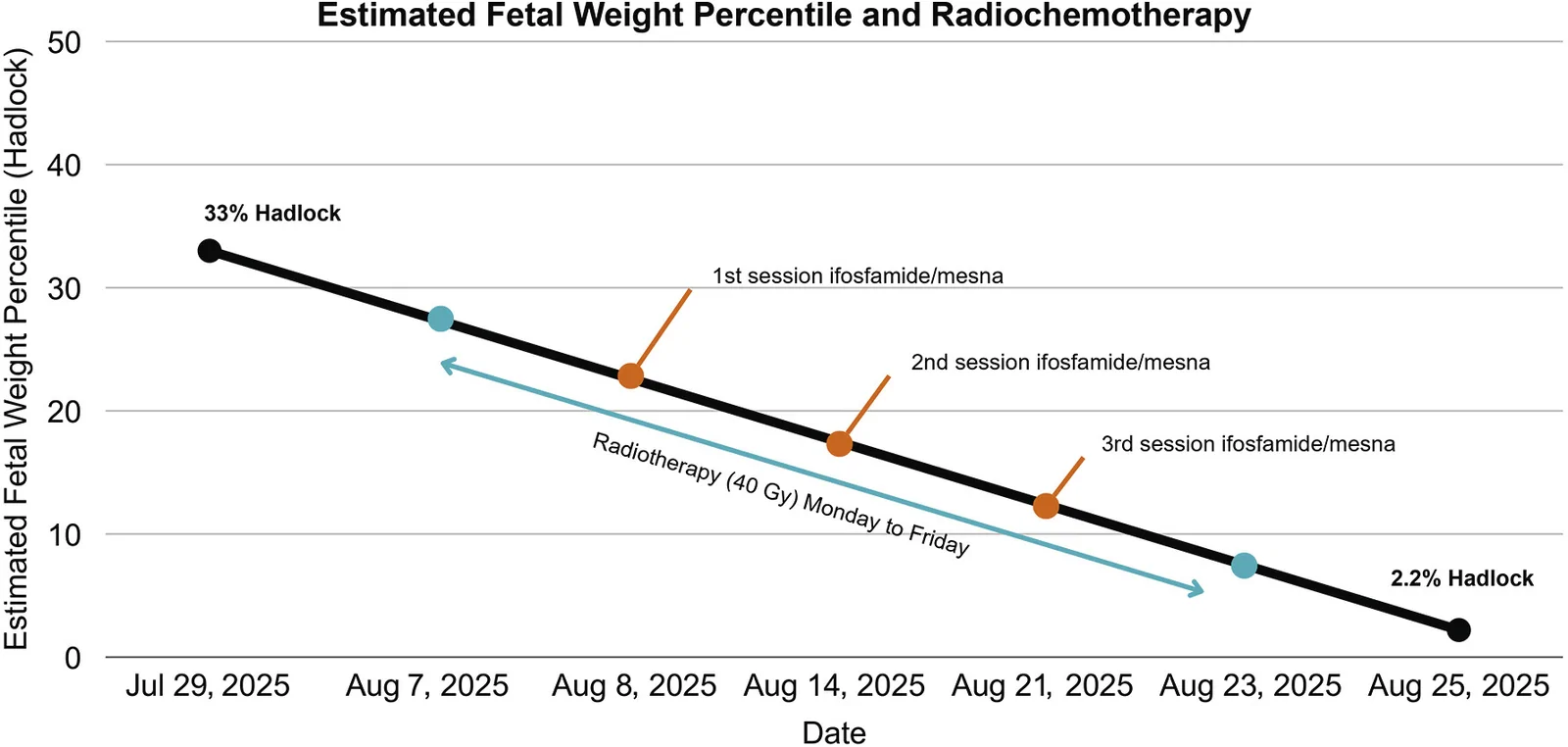
Estimated fetal weight (EFW) percentile and timing of radiochemotherapy. Black circles and line represent EFW percentiles according to Hadlock, blue markers indicate radiotherapy sessions (40 Gy, Monday through Friday), and orange markers indicate ifosfamide/mesna chemotherapy sessions.

**Fig. 2:**
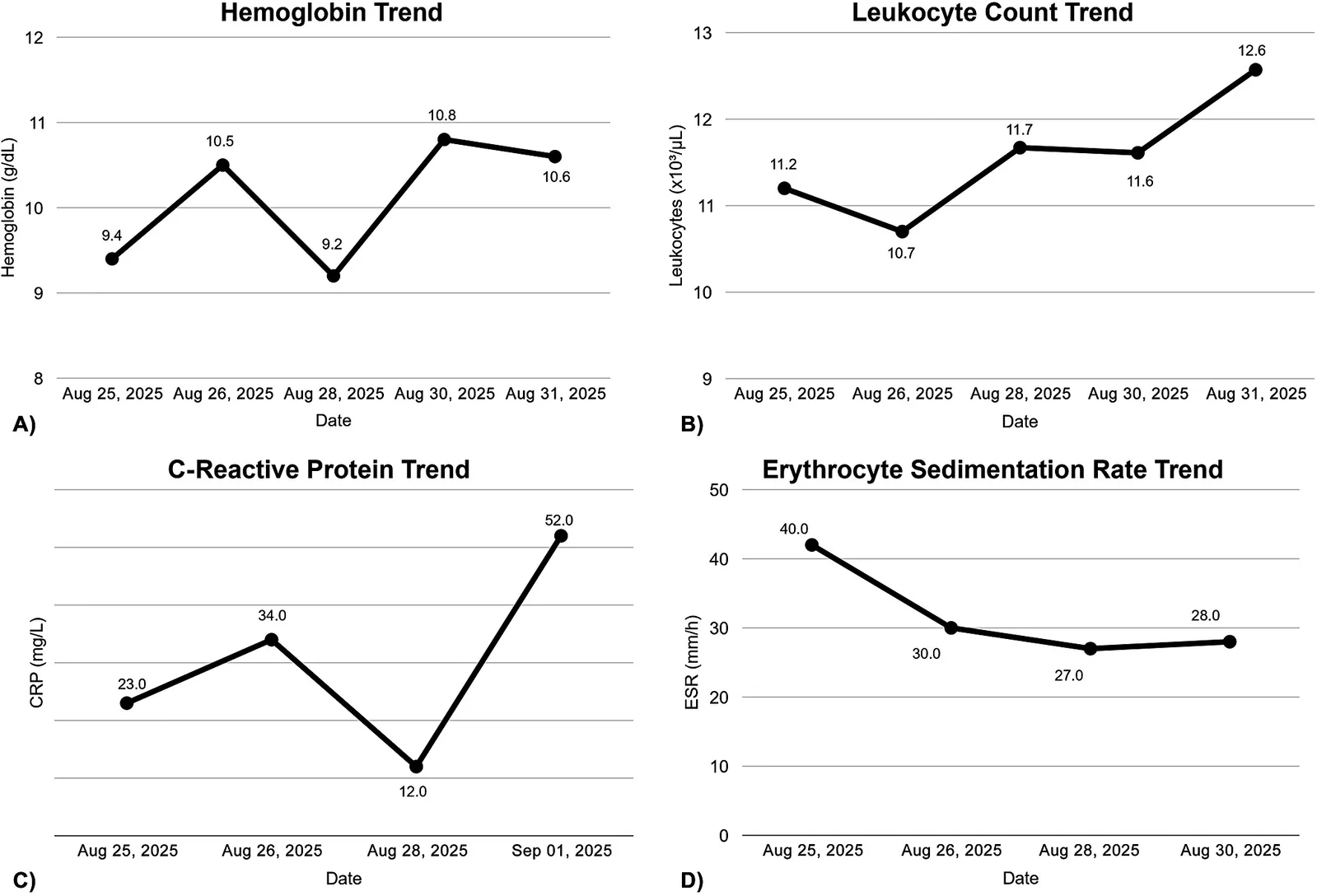
Laboratory findings. ( **A** ) Hemoglobin trend, ( **B** ) leukocyte count trend, ( **C** ) C-reactive protein (CRP) trend, and ( **D** ) erythrocyte sedimentation rate (ESR) trend.

**Fig. 3:**
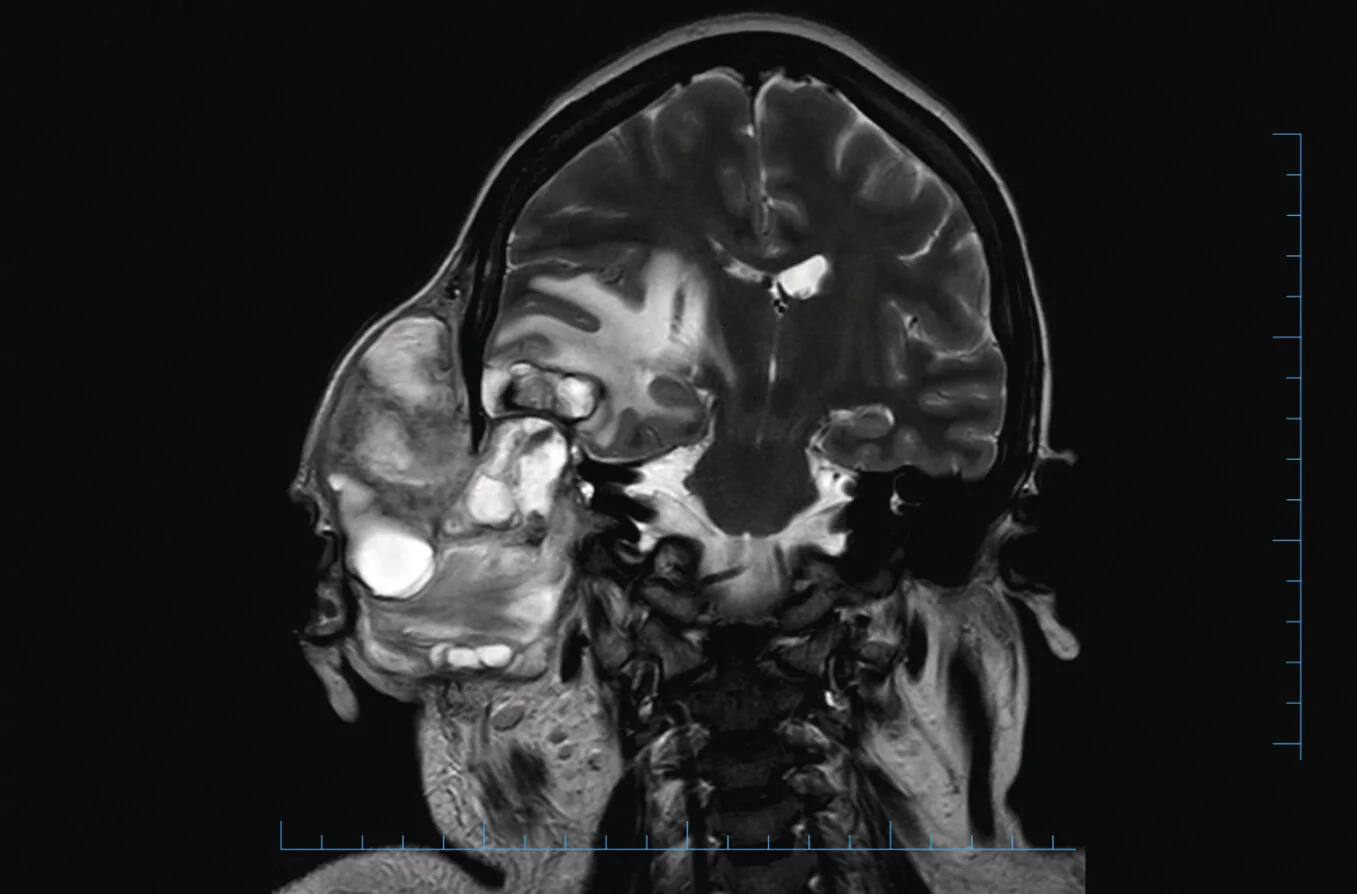
Coronal T2-weighed brain magnetic resonance imaging (MRI) with contrast.

**Fig. 4:**
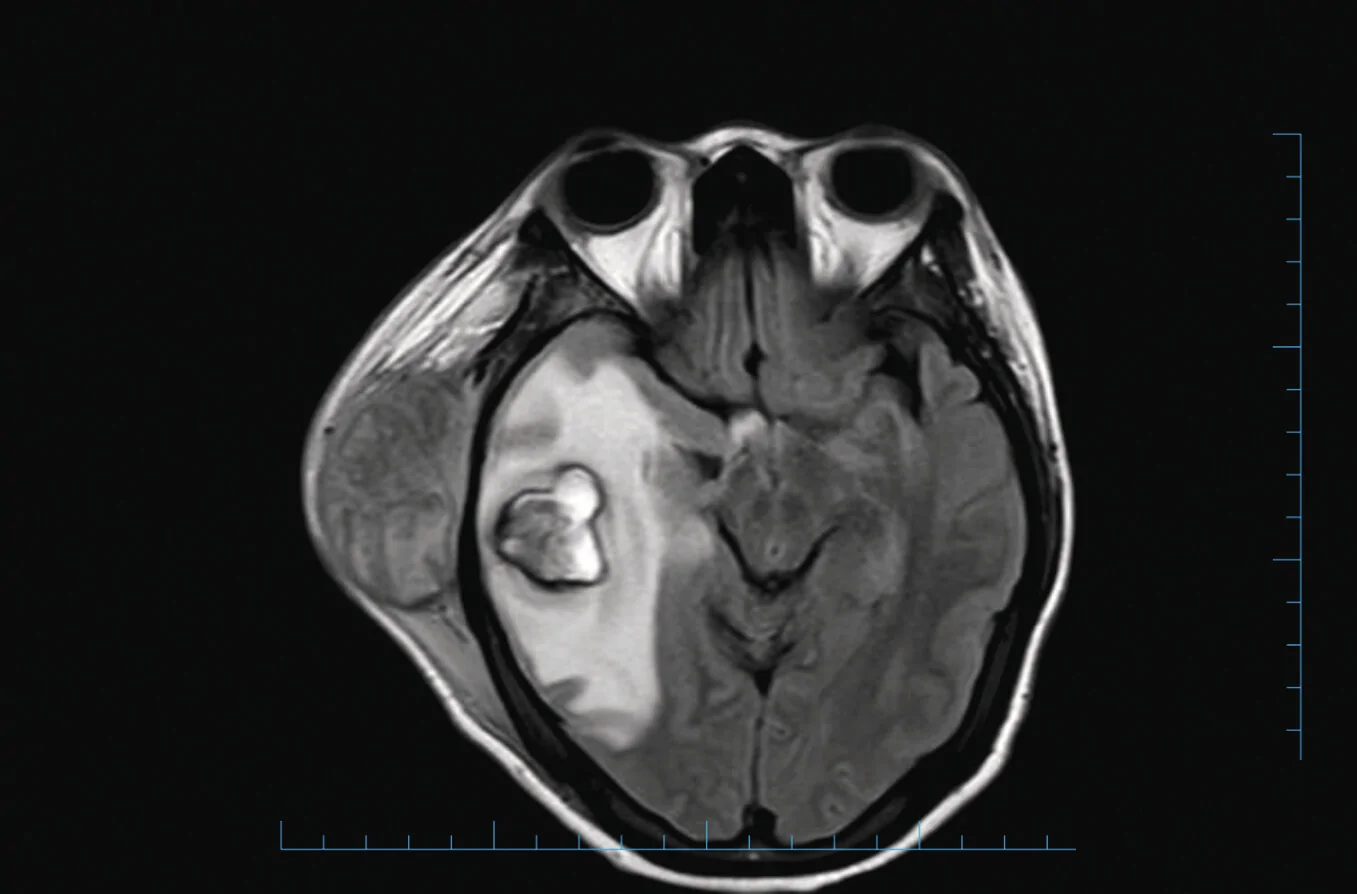
Axial T2-FLAIR brain magnetic resonance imaging (MRI) with contrast.

Pathological examination of the placenta is summarized in Table 1 , and representative histopathologic findings are shown in Figs. 5 and 6 .

**Table 1:** Placental pathology findings.

Placental pathology findings
Placenta: weight 445 g (small for gestational age) Umbilical cord: length 17.5 cm, three vessels present; mild diffuse edema (1.4 cm, >90th percentile), hypocoiled, with hypoplasia of umbilical vessels measuring 1 mm in diameter Freedman–Ernst phenotype: high-grade maternal vascular malperfusion with low-grade fetal vascular malperfusion (score 5) Decidua: moderate hypertrophic decidual vasculopathy with perivasculitis Chorionic plate: subacute thrombosis of one large chorionic vessel; <45 avascular villi; no karyorrhexis identified Parenchyma: placental infarctions involving ~15% of the parenchyma Villi: moderate increase in syncytial knots and perivillous fibrinoid deposition No atypical cells were identified within the intervillous space

**Fig. 5:**
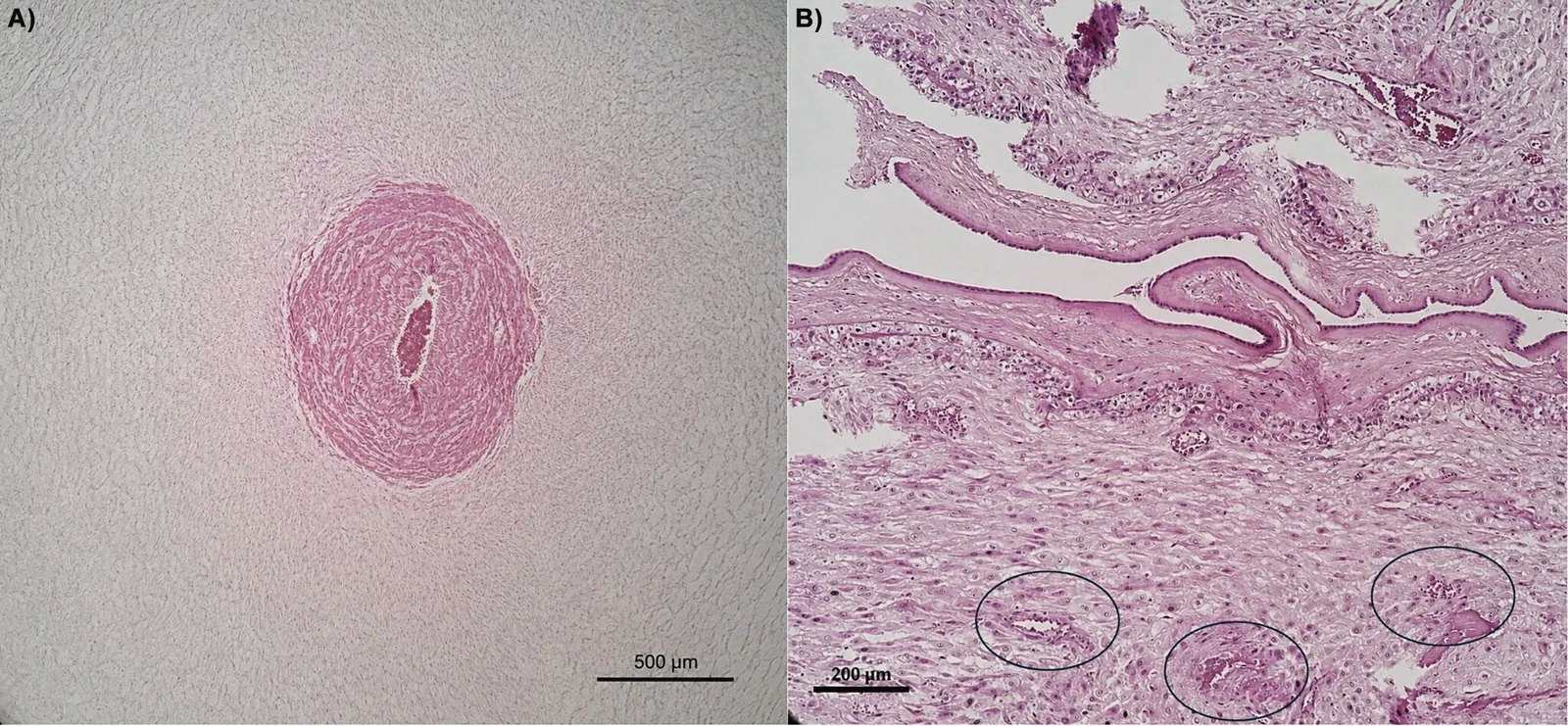
Histopathologic findings (hematoxylin and eosin staining): Umbilical cord and fetal membranes. ( **A** ) Panoramic view of an umbilical artery showing abundant Wharton’s jelly and reduced arterial caliber, with transverse measurements of 1025 × 875 μm, consistent with umbilical artery hypoplasia. ( **B** ) Fetal membranes. Circled decidual arteries show defective remodeling of the tunica media with mural thickening (mural hypertrophy) and perivascular lymphocytic infiltration consistent with perivasculitis.

**Fig. 6:**
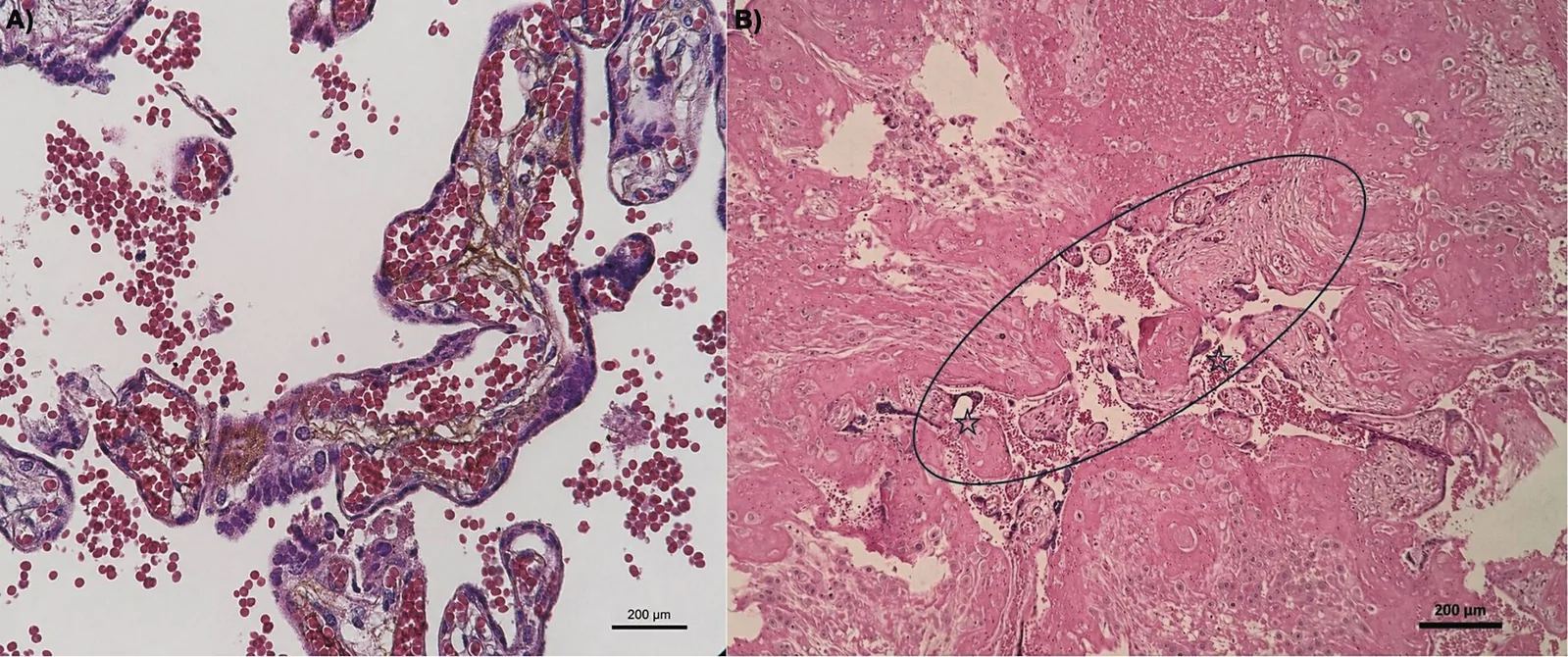
Histopathologic findings (hematoxylin and eosin staining): Placental parenchyma. ( **A** ) Large area of placental parenchyma devoid of chorionic villi. Within the villi, organized hemosiderin deposition (brown intravillous pigment), consistent with chronic hemorrhage. ( **B** ) Extensive ischemic infarction with necrosis of chorionic villi. Note the residual villi (oval) lacking stromal vessels, with prominent cytotrophoblasts and increased syncytial knots (stars).

The neonate remained hospitalized for 58 days, during which time abnormalities associated with maternal chemotherapy exposure were ruled out. He developed respiratory distress syndrome requiring one dose of surfactant and mechanical ventilation for 2 days, followed by nasal cannula oxygen therapy for 46 days. Vasoactive amines were not required, and cardiac function was adequate at discharge. He received pasteurized human milk and was managed with prokinetic agents. He developed non-hemolytic hyperbilirubinemia, for which he underwent 72 h of phototherapy. Serial laboratory testing showed no evidence of acute kidney injury or liver dysfunction. Renal function remained stable throughout hospitalization, as demonstrated by serial serum creatinine measurements, with the highest value recorded on day 6 of life (Cr: 0.59 mg/dL). The estimated glomerular filtration rate (eGFR), calculated using the Schwartz formula, was 19.85 mL/min/1.73 m ^2^ , which is within the expected range for a preterm neonate of this age. During hospitalization, he presented anemia associated with prematurity, requiring three packed red blood cell transfusions, without evidence of additional cytopenias or toxicity. No clinical signs of neurological injury were observed, and multiple cranial ultrasound examinations showed no evidence of hemorrhage.

## Discussion

As stated above, cancer during pregnancy is a delicate and distressing condition that occurs in 1:1000 pregnancies. 1 One of the main challenges in these cases lies in management and patient counseling. Although current guidelines may recommend pregnancy termination to allow for the administration of optimal chemotherapy or radiotherapy regimens in certain cancers, some patients choose to continue their pregnancies. This decision presents a significant clinical and ethical challenge, requiring the healthcare team to individualize treatment to maximize maternal benefit while minimizing fetal risk. 17

The main recommendations when it comes to the oncological treatment during pregnancy, as per international guidelines, are to carefully balance maternal and fetal well-being, shared decision-making, individualized care plans, and guidance from multidisciplinary expert teams, including medical oncologists, gynecological oncologists, surgical oncologists, radiation oncologists, radiologists, maternal–fetal medicine specialists, neonatologists, pediatricians, obstetricians, psychologists/psychiatrists as well as ethical and legal advisors. 17

Regarding fetal risk from exposure to chemotherapy and other oncologic treatment, this is generally related to the gestational period in which the therapy is received. Generally, it is of great importance to avoid delays in treatment, and thankfully, existing evidence and clinical practice guidelines suggest that the use of many chemotherapeutic agents is feasible and generally safe when initiated after 12–14 weeks of gestation (to avoid the organogenesis period) and prior to the third trimester. 17 18 19 It is recommended to avoid childbirth during periods of chemotherapy-induced hematologic nadir, characterized by neutropenia and thrombocytopenia. In accordance with the chemotherapy protocol, treatment should be discontinued 1–3 weeks before a predicted spontaneous delivery at term or before an elective delivery, whichever occurs earlier, and noting that premature delivery should be avoided when possible. 17 20 Nonetheless, certain chemotherapeutic agents are contraindicated during pregnancy, such as BEACOPP (bleomycin, etoposide, doxorubicin, cyclophosphamide, vincristine, procarbazine, and prednisone), high-dose alkylating agents (e.g., cyclophosphamide, busulfan, and melphalan), and methotrexate. 17 21 Concerning the mode of delivery, vaginal delivery is preferred unless obstetrically indicated. During the postpartum period, decisions regarding breastfeeding should involve multidisciplinary evaluation of maternal and neonatal safety due to the neonatal exposure to chemotherapeutic agents after delivery. 17 22

Although rare, sarcomas are seen with relative frequency in adolescents and young adults, which is why they may present in pregnancy, 23 24 most commonly soft tissue sarcomas such as leiomyosarcoma and liposarcoma although bone sarcomas such as osteosarcoma and Ewing’s sarcoma are also reported. 23 25

Anthracyclines are commonly included in standard therapeutic algorithms for sarcoma management. Data from studies of pregnancy-associated breast cancers and hematologic malignancies support the safety of anthracyclines (except idarubicin) when administered during the second and third trimesters. 23 26 However, sarcoma treatment guidelines usually add less well studied agents in pregnancy, such as ifosfamide. In the largest multi-institutional retrospective study to date examining the use of anthracyclines and/or ifosfamide during pregnancy-associated sarcomas, Miller et al reported outcomes for 13 pregnant patients, including 4 with bone sarcomas, three of which were osteosarcoma. All patients received anthracycline during pregnancy, most commonly doxorubicin at doses ranging from 60 to 75 mg/m ^2^ per cycle, and 69.2% received combination therapy with ifosfamide at doses of 7.5–10 g/m ^2^ per cycle. Overall, live birth occurred in only 69.2% of cases, with all pregnancies complicated by prematurity and a mean gestational age at delivery of 30.8 ± 3.8 weeks. Pregnancy complications were frequent, observed in 76.9% of patients, most commonly fetal growth restriction (46.2%), and two-thirds of neonates required NICU admission. Notably, all four cases of fetal demise occurred exclusively in patients receiving doxorubicin–ifosfamide combination therapy, whereas all patients treated with anthracycline-based regimens without ifosfamide experienced live births. Furthermore, chemotherapy initiation occurred significantly earlier among patients with pregnancy loss (mean: 15.5 weeks of gestation) compared with those with live births (mean: 21.3 weeks of gestation; *p* = 0.016). Higher cumulative ifosfamide exposure was also observed in pregnancies complicated by fetal demise, suggesting a potential dose-dependent effect. These findings raise concern that anthracycline–ifosfamide combination regimens, particularly when initiated early in the second trimester or administered at higher cumulative doses, may confer a substantially increased risk of adverse fetal outcomes compared with anthracycline monotherapy. 23

Ifosfamide is an oxazaphosphorine alkylating agent which inhibits DNA transcription and replication and is one of the pillars of the treatment of high-grade sarcomas, usually in combination with anthracycline. This drug may present with non-specific adverse effects such as pancytopenia and mucositis, as well as more specific adverse effects like neuro- and nephrotoxicity. Devant et al performed a review of 27 cases to determine the safety of ifosfamide in pregnancy. No congenital malformations were identified; however, fetal growth restriction (56%), oligohydramnios/anhydramnios (56%), and fetal death (19%) were found. It is important to note that all fetal death cases were exposed to ifosfamide before the 21st week of gestation. All 22 of the live births were preterm, and acute renal failure in the newborn was present in 23% of the cases. Neonatal death occurred in 14% of all live births due to anuria. These findings underline the importance of serial fetal ultrasound surveillance in pregnant patients undergoing chemotherapy with ifosfamide to allow for the early detection of fetal nephrotoxicity and oligohydramnios, allowing timely intervention and optimization of perinatal outcomes. 16

The case presented in this report closely reflects the complexities and risks outlined in the literature regarding chemotherapy during pregnancy. The multidisciplinary decision to continue the pregnancy while administering chemoradiation was guided by current recommendations for individualized care and shared decision-making. Notably, the adverse fetal outcomes observed in this case, including early-onset fetal growth restriction and anhydramnios, are consistent with published data indicating that exposure to ifosfamide between 16 and 30 weeks of gestation is associated with fetal nephrotoxicity and growth impairment. Furthermore, recent studies have shown that combination regimens involving anthracyclines and ifosfamide, especially when initiated early in the second trimester or at higher cumulative doses, are linked to increased rates of fetal demise, prematurity, and neonatal complications compared with anthracycline monotherapy. The neonatal outcomes in this case, including the need for NICU admission, reinforce these findings and highlight the importance of careful risk assessment and ongoing research to guide the management of rare cancers in pregnancy.

## Data Availability

The data supporting the findings of this case report are not publicly available due to patient privacy and institutional restrictions. No dataset was generated or deposited for this study. Relevant data may be available from the corresponding author upon reasonable request and subject to institutional approval.

## Declaration of GenAI Use

ChatGPT (OpenAI) was used during manuscript preparation to improve grammar and wording in select paragraphs. The authors reviewed and approved all AI-assisted text and remain fully responsible for the content of the manuscript.
